# The benefits of influenza vaccination in patients with cardiovascular disease: a systematic review and meta-analysis

**DOI:** 10.3389/fphar.2025.1701127

**Published:** 2026-01-20

**Authors:** Meng Wei, Lei Liu, Kaiyu Zhou, Yifei Li

**Affiliations:** Key Laboratory of Birth Defects and Related Diseases of Women and Children of MOE, Department of Pediatrics, NHC Key Laboratory of Chronobiology, West China Second University Hospital, Sichuan University, Chengdu, Sichuan, China

**Keywords:** cardiovascular diseases, high-dose influenza vaccination, influenza vaccine, major adverse clinical events, mortality

## Abstract

**Background:**

The clinical impact of high-dose influenza vaccination on cardiovascular outcomes in patients with established cardiovascular disease (CVD) remains controversial. To this end, our study aimed to assess the impact of influenza and high-dose influenza vaccine on cardiovascular outcomes in patients with CVD or at high risk for CVD.

**Methods:**

We systematically searched three electronic databases from construction to 5 November 2025. The data extraction and meta-analysis were conducted following PRISMA workflow, utilizing both fixed- and random-effects models to ensure robust statistical analysis. The primary clinical outcomes included all-cause mortality (ACM) and major adverse clinical events (MACEs). Secondary endpoints included all-cause hospitalization (ACH), cardiovascular mortality (CVM), heart failure (HF), myocardial infarction (MI), stroke, ICU admission, and non-cardiovascular death.

**Results:**

A total of 36 studies were included for this research. According to the scope of the research, hierarchical analyses were performed among several subgroups. For efficacy evaluation, we included 25 articles, with a total of 869,795 vaccinated and 1,306,470 unvaccinated participants. Influenza vaccination was associated with a significant reduction in ACM (OR = 0.74; 95% CI, 0.60–0.91), MACE (OR = 0.62; 95% CI, 0.46–0.83), and ICU admission (OR = 0.33; 95% CI, 0.22–0.49) compared to those in the control group. Influenza vaccine only provided a significant prevention of CVM (OR = 0.59; 95% CI, 0.39–0.89) and ACH (OR = 0.80; 95% CI, 0.74–0.87) among established CVD patients. No significant advantage of influenza vaccine in reducing the incidence of HF, non-cardiovascular death, MI, and stroke was observed. Then we also evaluated the efficacy of high-dose vaccination strategy in CVD management. Eleven studies were included for this purpose, and the pooled outcome analysis demonstrated that the high-dose strategy did not provide any benefit in reducing ACM and MACE.

**Conclusion:**

The influenza vaccination provided significant benefits in reducing ACM and MACE. However, it failed to demonstrate advantage in managing HF, MI, and non-cardiovascular death in patients with CVD. Additionally, the high-dose influenza vaccination strategy did not present efficacy in preventing adverse outcomes of CVD compared to the standard strategy.

## 1 Introduction

Influenza viruses are enveloped, segmented, negative-sense, single-stranded RNA viruse**s** belonging to the family Orthomyxoviridae and are classified into four types (A, B, C, and D) based on their nucleoprotein and matrix protein antigens. Influenza causes an estimated 1 billion illnesses globally each year, leading to 3 to 5 million severe cases. Cardiovascular diseases (CVDs) remain the leading cause of death worldwide, accounting for approximately 17.9 million deaths (32% of all deaths) (WHO, 2025). Multiple epidemiologic and clinical studies have shown that influenza infection is associated with an increased risk of adverse cardiovascular outcomes in patients with underlying CVDs (Kwong et al., 2018; Ohland et al., 2020; Warren-Gash et al., 2012; Skhi et al., 2012; Teutsch et al., 2021). Furthermore, clinical studies have shown that the incidence of acute myocardial infarction (MI) and ischemic stroke reached a significant peak within 1–3 days after laboratory-confirmed influenza infection (Warren-Gash et al., 2012). Chotpitayasun et al. reported 12 patients with confirmed influenza infection, five of whom developed concomitant heart failure (HF); all subsequently died within 8–21 days after infection (Chotpitayasun et al., 2005). According to the few reports available, fatal arrhythmias often occur early in the disease, usually within 24 h after influenza infection (Estabragh and Mamas, 2013), and overall CVD deaths peaked within 21 days of laboratory-confirmed influenza infection (Nguyen et al., 2016). The proposed mechanisms linking influenza infection to adverse cardiovascular outcomes include systemic inflammation leading to destabilization and rupture of atherosclerotic plaques with subsequent thrombosis, immune complex deposition within plaques, endothelial dysfunction, and recruitment and activation of macrophages within the arterial wall (Conti, 1993; Hebsur et al., 2014; Kinlay and Ganz, 1997). Influenza vaccination may confer immediate cardioprotection in part by preventing the trigger of cardiovascular events mediated by plaque destabilization and thrombosis (Corrales-Medina et al., 2011; Ciszewski, 2018).

Accumulating evidence suggests that influenza vaccination reduces the incidence of cardiovascular events in high-risk populations (Udell et al., 2013; Yedlapati et al., 2021). However, several studies indicate that patients with CVD may elicit weaker and less durable humoral responses to standard-dose influenza vaccines than healthy individuals (Vardeny et al., 2009; Naruse et al., 2021; Albrecht et al., 2014), raising concerns over suboptimal protection. High-dose formulations, which contain increased hemagglutinin antigen content, may enhance immunogenicity and clinical effectiveness in this population. Therefore, we conducted a systematic review and meta-analysis to evaluate the protective efficacy of influenza vaccination in providing secondary prevention against adverse outcomes among patients with established CVD. Additionally, we evaluated the efficacy of high-dose vaccination strategy in CVD management compared to the standard dose.

## 2 Materials and methods

### 2.1 Study protocol

This systemic review was conducted according to the Preferred Reporting Items for Systemic Reviews and Meta-Analysis (PRISMA) guidelines for the quality of reporting of meta-analysis. Our study protocol was registered on PROSPERO (CRD420251079983).

### 2.2 Search strategy

A systematic literature search was performed in PubMed, Embase, and Scopus databases from inception through 5 November 2025 using a comprehensive search strategy developed in collaboration with a medical librarian (Supplementary Table S1). The search terms included “influenza vaccination,” “cardiovascular diseases,” and related keywords combined with Medical Subject Headings (MeSH) terms. The search strategy was designed to identify all randomized controlled trials (RCTs) and cohort interventional studies evaluating influenza vaccination for the prevention of adverse cardiovascular outcomes in patients with established CVD. No restrictions were applied for publication year or language. We manually reviewed reference lists of included studies and relevant systematic reviews to identify additional eligible publications. Complete search strategies for all databases are provided in Supplementary Table S1.

### 2.3 Study selection

Titles and abstracts of search results were screened independently (YL). The full texts of the remaining results were assessed independently by two reviewers (MW and LL) for inclusion based on predetermined criteria. Any discrepancies were resolved through discussion, potentially with a third reviewer. In addition, we manually screened the reference lists of retrieved articles and pertinent literature reviews, along with all articles citing the included studies.

Our study inclusion criteria according to the PICOS framework were (1) Population: the study population includes patients with all-cause-induced established CVDs, including acute coronary syndrome (ACS), HF, any type of arrhythmias, cardiomyopathy, and congenital heart diseases. In addition, the study included patients with a relatively high risk of CVD, including those with hypertension, diabetes mellitus, aging-related diseases, disability, and chronic renal disease with a minimum of 12 months of follow-up. (2) Intervention: influenza vaccination administered at a high dose (double or quadruple the standard dose of vaccine) or standard dose. (3) Comparison intervention: CVD patients who failed to receive influenza vaccine or received a placebo. In dose evaluation, the comparison was set as established CVD patients who received standard-dose influenza vaccine. (4) Outcome: for the effective evaluation of influenza vaccine in the study, we considered major adverse cardiovascular events (MACEs), all-cause mortality (ACM), all-cause hospitalization (ACH) rate, cardiovascular disease mortality (CVM), HF, MI, stroke, and ICU hospitalization rate. For the evaluation of efficacy of different vaccine doses, the incidence of MACE, ACM, ACH, CVD hospitalization or mortality, cardiopulmonary disease, HF, heart infarction, stroke, unstable angina, pneumonia, and respiratory disease was implicated (5) Study design: RCTs or interventional cohort studies.

The study exclusion criteria were (1) the administration of multiple vaccines, such as a combination of pneumococcal and influenza vaccines; (2) case report, case–control study, review, or abstract; (3) animal studies; (4) duplicate publications; and (5) studies lacking relevant outcome.

### 2.4 Data extraction and assessment of study quality

The relevant articles and eligible data were assessed and extracted by two authors (MW and LL), respectively. Disagreements were resolved by consensus with the third author (YL). We used the GRADE criteria to assess the quality of evidence for each study.

The following data were collected from each study: first author name, area, publication date, study design, number of patients, comparison intervention and primary outcome (ACM and MACE), and secondary outcomes (ACH, CVM, etc.). All data were presented as categorical variables; any data originally presented in other formats were converted into categorical variables based on ratio or incidence calculation. Table 1 presents the baseline characteristics of the included studies.

**TABLE 1:** Main characteristics of the included studies on the evaluation of vaccine efficacy.

Study	Duration of the study	Region	Study design	Population description	Mean age	Sex	Relevant outcome
Anderson et al. (2025)	2021–2024	China	RCT	Patients with HF	Unvaccinated: 72: vaccinated: 71.7	47.1% female	ACM and ACH
Miró et al. (2025)	2022.11–2022.12	Spain	Observational study	Patients with HF	85	56.6% female	ACM and ACH
Miró et al. (2023)	2018–2019	Spain	Observational study	Patients with HF	84	56% female	ACM and ACH
Modin et al. (2022)	2007–2016	Denmark	Observational study	Patients with hypertension	65.2	43.8% male	ACM, CVM, MI, and stroke
Mefford et al. (2022)	2009–2017	United States	Observational study	Patients with HF	Unvaccinated: 71.6; vaccinated: 71.9	55.3% male	ACM, CVM, and ACH
Loeb et al. (2022)	2015.6.2–2021.11.21	India, etc.	RCT	Patients with HF	57.2	48·6% male	ACM, MACE, CVM, ACH, HF, and stroke
Fröbert et al. (2021)	2016.10.1–2020.3.1	Sweden, etc.	RCT	Patients with MI	59.9	18.2% female	ACM, MACE, CVM, HF, MI, and stroke
Modin et al. (2020)	2007–2016	Denmark	Observational study	Patients with diabetes	58.7	52.9% male	ACM and CVM
Chang et al. (2020)	2014.1.1–2015.9.30	Taiwan	Observational study	Patients with disabilities	N/A	N/A	ACM and MACE
Wu et al. (2019)	2000–2013	Taiwan	Observational study	Patients with MI	Vaccinated: 76.3; unvaccinated: 76	Vaccinated cohort: 65.83% male; unvaccinated cohort: 61.95% male	ACM, MACE, CVM, HF, and MI
Christiansen et al. (2019)	2005.1.1–2015.12.31	Denmark	Observational study	Patients hospitalized in Danish ICUs	N/A	56.9% male	ACM, HF, MI, and stroke
Liu et al. (2017)	2005.1.1–2012.12.31	Taiwan	Observational study	Patients with atrial fibrillation	73.39	52.82% male	Stroke
Kaya et al. (2017)	2013.12; 2014.12	Turkey	Observational study	Patients with HF	62	28% female	HF
Vamos et al. (2016)	2003.4–2009.10	England	Observational study	Patients with type 2 diabetes	66.2	Vaccinated: 53.9% male	ACM, HF, MI, and stroke
Fang et al. (2016)	1999.1.1–2008.12.31	Taiwan	Observational study	Patients with CKD	N/A	Vaccinated: 60.78% male; unvaccinated: 55.81% male	HF
Wang et al. (2013)	1998–2009	Taiwan	Observational study	Patients with end-stage renal disease	Vaccinated: 70.2; unvaccinated: 59.4	Vaccinated: 50.3% male; unvaccinated: 48.7% male	ACM, MACE, and ACH
Liu et al. (2012)	1997.1–2002.9	Taiwan	Observational study	Patients with IHD	75.2	Vaccinated: 58.3% male; unvaccinated: 51.8% male	ACM and MACE
Johnstone et al. (2012)	2003–2007	40 countries	Observational study	Patients with vascular disease or diabetes	N/A	N/A	MACE
Phrommintikul et al. (2011)	2007.11–2008.10	Thailand	RCT	Patients with ACS	66	Vaccinated: 61% male; unvaccinated: 52% male	ACM, MACE, HF, and stroke
de Diego et al. (2009)	2002.1–2005.4	Spain	Observational study	Patients with heart failure or CAD	76.2	47.4% male	ACM
Ciszewski et al. (2008)	2004.10–2005.12	United States	RCT	Patients with CAD	59.9	72.5% male	MACE, CVM, and MI
Wang et al. (2007)	N/A	Taiwan	Observational study	High-risk patients and low-risk patients	Vaccinated cohort: 38.1% (≥ 75 years); unvaccinated cohort: 39.8% (≥ 75 years)	54.6% male	ACM
Wang et al. (2004)	2001.1.1–2001.6.30	Taiwan	Observational study	Elderly persons (age ≥65 years)	34% (≥75 years)	N/A	ACH
Gurfinkel et al. (2004)	N/A	Argentina	RCT	Patients with MI	N/A	N/A	MACE, CVM, ACH, and MI
Gurfinkel et al. (2002)	N/A	Argentina	RCT	Patients with MI	Vaccinated: 64; unvaccinated: 63	N/A	ACM, MACE, and CVM

CVD, cardiovascular diseases; ACH, all-cause hospitalization; ACM, all-cause mortality; CVM, cardiovascular mortality; HF, heart failure; MI, myocardial infarction; MACE, major adverse cardiac events containing history of sustained ventricular tachycardia, ICD, appropriate therapy, and syncope.

Two investigators (MW and LL) independently appraised the potential risk of bias using the Cochrane risk of bias tool for RCTs (see Supplementary Figure S1) and the Newcastle–Ottawa Scale (NOS) for cohort studies (Supplementary Table S2). We then classified the studies as low quality, unclear, or high quality based on the scores obtained after evaluation. We extracted the following information: first author’s name, year of publication, duration of follow-up, country, sample size, population, age, sex, type of influenza vaccine, injection strategy, and primary/secondary outcomes.

### 2.5 Statistical analysis

The meta-analysis used the combined effects of each result. Binary outcomes were analyzed using odds ratios (ORs) with 95% confidence intervals (CIs), and both were derived using random-effects models to account for potential heterogeneity among studies. We applied funnel plots and Egger’s test to assess publication bias. All meta-analyses (127) were conducted using the meta package in R 4.4.3 software (www.r-project.org). A two-sided p-value of 0.05 was deemed statistically significant.

The results of this study were presented in forest plots. Each horizontal line segment in the forest plot represents the OR and its 95% CI from an individual study. The size of the square at the center of each segment is proportional to the study’s weight in the meta-analysis. The diamond at the bottom of the plot represents the pooled effect estimate and its 95% CI across all included studies. The vertical line of no effect corresponds to an x-axis value of 1 and is used to assess statistical significance. If a study’s CI crosses this line, its effect is not statistically significant. Similarly, if the pooled effect’s CI crosses this line, the overall pooled effect is not statistically significant.

## 3 Results

**FIGURE 1:**
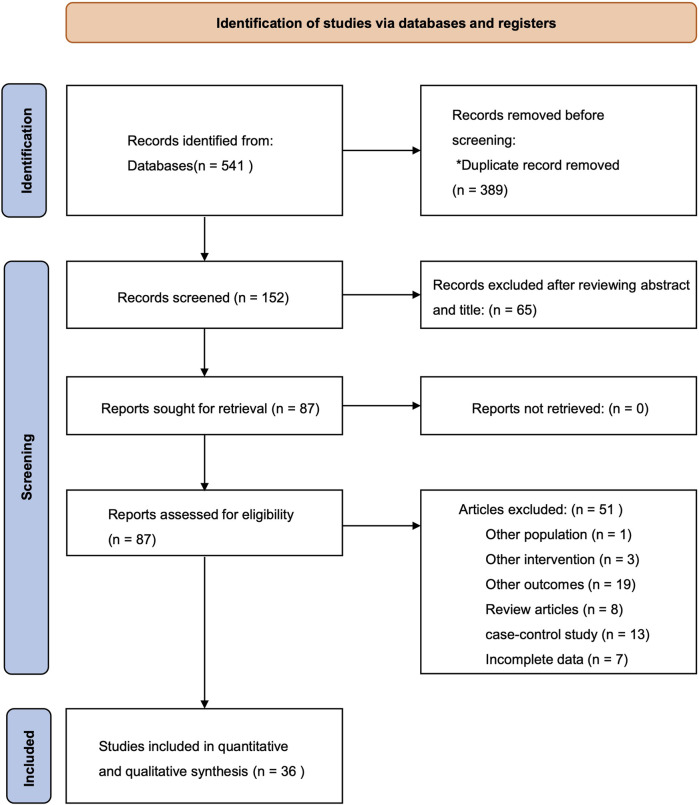
Preferred Reporting Items for Systematic Review and Meta-analysis (PRISMA) flow of the search strategy for systematic review and meta-analysis.

Our initial search identified 541 articles. After removing 389 duplicates, 65 studies were excluded after a review of their titles and abstracts. A total of 87 studies were reviewed in full-text form; however, 51 studies were further excluded based on inclusion criteria. A total of 36 studies that met our specified criteria were ultimately selected for data analysis and system review: 25 studies on vaccine efficacy and 11 studies on vaccine dosing (Figure 1).

### 3.1 Efficacy and effectiveness of influenza vaccination in reducing adverse outcomes among patients with CVD

We pooled data from 25 studies on the prevention of adverse cardiovascular events by influenza vaccine (Tables 1, 2). The effectiveness analysis included a total of 869,795 vaccinated and 1,306,470 unvaccinated participants.

**TABLE 2:** Main characteristics of the included studies on vaccination strategies.

Study	Duration of the study	Country	Study design	Population description	Mean age	Sex	Relevant outcome
Pareek et al. (2025)	2022–2025	Denmark	RCT	Patients with ASCVD	75.2	66.5% male	ACM, CVM, ACH, MACE, HF, MI, stroke, hospitalization for respiratory disease, and hospitalization for cardiorespiratory disease
Johansen et al. (2025a)	2022–2025	Denmark	RCT	Residents aged 65 years or older	73.7	51.4% male	ACH, MACE, hospitalization for respiratory disease, and hospitalization for cardiorespiratory disease
Johansen et al. (2025b)	2022–2025	Denmark	RCT	Residents aged 65 years or older	75.3	63.4% male	CVM, ACH, MACE, stroke, hospitalization for respiratory disease, and hospitalization for cardiorespiratory disease
Christensen et al. (2025)	2021–2022	Denmark	RCT	Patients with chronic CVD	71.7	52.9% male	ACM, CVM, ACH, and hospitalization for respiratory disease
Peikert et al. (2024)	2016.9–2019.1	United States and Canada	RCT	Patients with MI or HF	Standard-dose quadrivalent vaccine: 60.1% (≥ 65 years); high-dose trivalent vaccine: 55.6% (≥ 65 years)	Standard-dose quadrivalent vaccine: 74.7% male; high-dose trivalent vaccine: 79.5% male	ACM and ACH
NajafZadeh et al. (2024)	2016–2019	United States	Observational study	Patients with HF	>65	57% female	ACM and ACH
Fonseca et al. (2024)	2019.7–2020.11	Brazilian	RCT	Patients with ACS	56.7	70% male	ACM, CVM, hospitalization for cardiorespiratory disease, hospitalization for unstable angina, MI, and stroke
Christensen et al. (2024)	2021.10.1–2021.11.20	Denmark	RCT	Participants aged between 65 and 79 years regardless of medical history	71.7	47.1% female	ACM
Saade et al. (2022)	2013.11.1–2014.5.31	United States	RCT	Long-stay residents aged 65 years or older	N/A	N/A	MACE
Fonseca et al. (2022)	2019.7.19–2020.11.30	Brazil	RCT	Patients with ACS	56.7	70% male	ACM, MACE, CVM, hospitalization for cardiorespiratory disease, hospitalization for respiratory disease, hospitalization for unstable angina, MI, and stroke
Vardeny et al. (2021)	2016.9.21–2019.1.31	United States and Canada	RCT	Patients with MI	​	​	ACM, MACE, and CVM

CVD, cardiovascular diseases; ACH, all-cause hospitalization; ACM, all-cause mortality; CVM, cardiovascular mortality; HF, heart failure; MI, myocardial infarction; MACE, major adverse cardiac events containing history of sustained ventricular tachycardia, ICD, appropriate therapy, and syncope.

**FIGURE 2:**
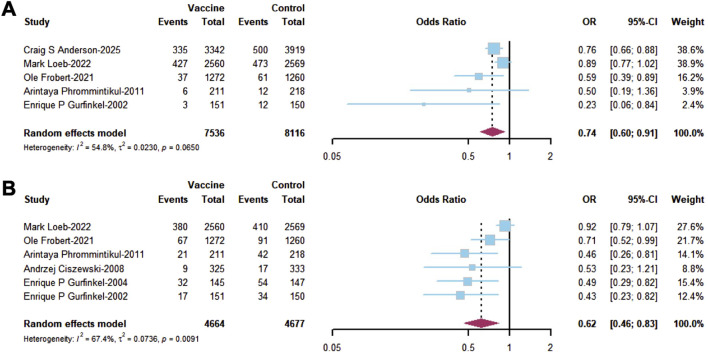
Forest plots of major outcomes in RCTs including **(A)** all-cause mortality (ACM) and **(B)** major adverse clinical events (MACEs).

The pooled analysis of primary outcomes showed that influenza vaccination was associated with a significant reduction in ACM (OR = 0.74; 95% CI, 0.60–0.91; I^2^ = 54.8%, Figure 2A) and MACE (OR = 0.62; 95% CI, 0.46–0.83; I^2^ = 67.4%, Figure 2B) compared to those in the placebo group in RCTs. A combined analysis of RCTs and cohort studies also demonstrated a significant reduction in ACM (OR = 0.75; 95% CI, 0.59–0.95; I^2^ = 99.6%, Supplementary Figure S2A). However, no significant reduction in MACE was observed based on the combined analysis of RCTs and cohort studies (OR = 0.84; 95% CI, 0.68–1.03; I^2^ = 82.6%, Supplementary Figure S2B).

**FIGURE 3:**
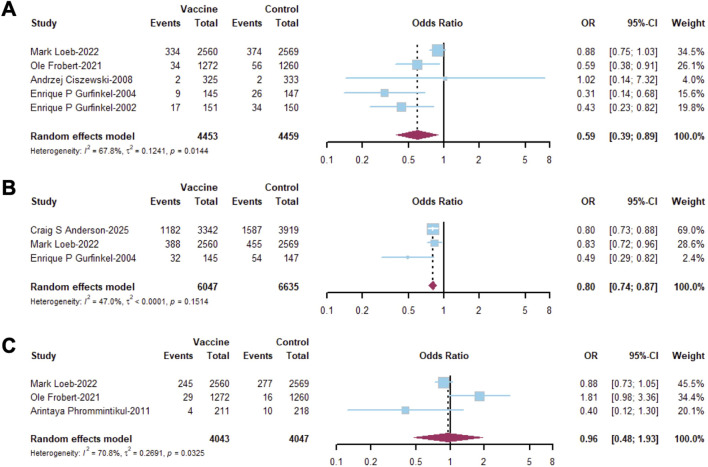
Forest plot of secondary outcomes in RCTs among patients with established CVD including **(A)** cardiovascular mortality (CVM), **(B)** all-cause hospitalization (ACH), and **(C)** heart failure (HF).

**FIGURE 4:**
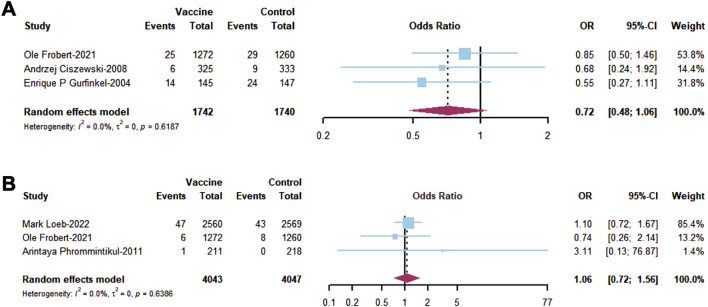
Forest plot of secondary outcomes in RCTs including **(A)** MI and **(B)** stroke.

In the evaluation of secondary outcomes based on RCTs, influenza vaccine provided a significant prevention of CVM (OR = 0.59; 95% CI, 0.39–0.89; I^2^ = 67.8%, Figure 3A) and ACH (OR = 0.80; 95% CI, 0.74–0.87; I^2^ = 47.0%, Figure 3B) among established CVD patients. No significant difference in HF (OR = 0.96; 95% CI, 0.48–1.93; I^2^ = 70.8%, Figure 3C) was observed between established CVD patients who received the influenza vaccine and the unprotected population. Unfortunately, the pooled results from RCTs and cohort studies did not demonstrate any advantages of influenza vaccine in managing secondary outcomes (Supplementary Figure S3). In addition, there was no significant reduction in the incidence of MI (OR = 0.72; 95% CI, 0.48–1.06; I^2^ = 0%, Figure 4A) and stroke (OR = 1.06; 95% CI, 0.72–1.56; I^2^ = 0%, Figure 4B) after influenza vaccine administration. Consequently, in a mixed pooled analysis of RCTs and cohort studies, influenza vaccination did not reduce the risk of MI and stroke among established CVD patients (Supplementary Figure S4).

### 3.2 Appropriate dosage administration of influenza vaccination in CVD management

**FIGURE 5:**
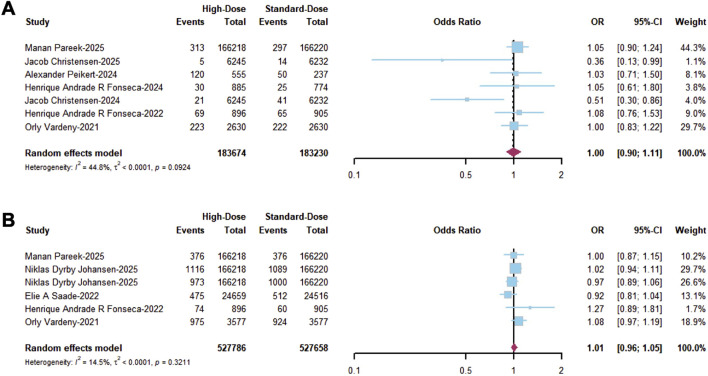
Forest plot of primary outcomes in RCTs including **(A)** ACM and **(B)** MACE.

A total of 210,181 high-dose and 209,591 standard-dose vaccine recipients were included in the efficacy analysis of vaccination dose, and only RCTs were included. The primary outcome analysis revealed that the high-dose influenza vaccination did not provide better efficacy in reducing ACM (OR = 1.00; 95% CI, 0.90–1.11; I^2^ = 44.8%, Figure 5A) and MACE (OR = 1.01; 95% CI, 0.96–1.05; I^2^ = 14.5%, Figure 5B) than standard-dose influenza vaccination. In the secondary outcome evaluation, no significant difference between high-dose and standard-dose influenza vaccination strategies in managing clinical outcomes of CVD patients, including CVM, incidence of all-cause hospitalization, hospitalization for cardiorespiratory disease, hospitalization for respiratory disease, hospitalization for unstable angina, MI, and stroke, was observed (Supplementary Figure S5). Therefore, these findings indicate that the high-dose influenza vaccine did not provide any additional protection compared to the standard dose, suggesting that the administration of the standard protocol would be comparatively efficient.

### 3.3 Publication bias and sensitivity analysis

No publication bias was identified using Egger’s test in the evaluation of the benefits of influenza vaccination in reducing particular clinical outcomes in patients with established CVDs, including ACM (p = 0.20), ACH (p = 0.68), CVM (p = 0.07), HF (p = 0.19), and MACE (p = 0.31) (Supplementary Figure S6). Due to the high heterogeneity, sensitivity analyses were performed for ACM, MACE, and CVM. These analyses demonstrated significant results, suggesting that influenza vaccination protects patients with established CVD by reducing mortality and MACE. Upon exclusion of one study at a time, the results consistently showed a significant reduction in ACM and MACE after influenza vaccine administration among patients with established CVD (Supplementary Figure S7).

## 4 Discussion

Previous studies showed that patients with established CVD develop forms of immunodeficiency, particularly in advanced stages. Thus, the administration of infection-associated vaccines is recommended in some guidelines and scientific statements. In this context, influenza vaccination administration strategies have been reported in some published studies. Patients with established CVD would often benefit from regular influenza vaccination. However, the details of the administration strategies and dosage consideration remain controversial (Addario et al., 2023; Jaiswal et al., 2022; Gupta et al., 2022; Diaz-Arocutipa et al., 2022; Rodrigues et al., 2020; Bhugra et al., 2021). To this end, we performed this meta-analysis to evaluate the efficacy of influenza vaccine in reducing specific adverse clinical outcomes and the advantages of high-dose vaccination. To address the knowledge gaps in the existing literature, we synthesized data from recently published large-scale RCTs and observational studies with earlier investigations. For the evaluation of vaccine effectiveness, we included 25 studies (7 RCTs and 18 observational studies), and 11 studies (11 RCTs) were analyzed for dose comparison. Our principal findings were as follows: (1) influenza vaccination significantly reduced ACM, MACE, CVM, and ACH in patients with established CVD and in those at high cardiovascular risk. The protective effect was more pronounced in RCTs than in observational studies and in patients with established CVD than in those at a high risk for CVD. (2) High-dose influenza vaccination did not demonstrate superior efficacy over standard-dose vaccination for preventing adverse cardiovascular outcomes in patients with CVD or in those at high cardiovascular risk.

Several other research groups have evaluated the cardioprotective effects of influenza vaccination. Jaiswal et al. (2022) conducted a meta-analysis of 18 studies (5 RCTs and 13 observational studies) that involved high-risk CVD populations and found that influenza vaccination significantly reduced ACM, MACE, and CVM, which was consistent with our results. However, their analysis demonstrated a significant reduction in MI risk and no benefit for HF outcomes, which contrasts with our findings for these endpoints. Our finding of no significant reduction in MI risk is supported by two other meta-analyses (Yedlapati et al., 2021; Diaz-Arocutipa et al., 2022). The discrepancy in MI estimates may be attributed to the relatively low incidence of this outcome compared with that of other cardiovascular events, leading to insufficient statistical power to detect modest treatment effects. Although epidemiologic studies have established an association between influenza infection and increased stroke risk, some previous meta-analyses demonstrated a significant association between influenza vaccination and stroke prevention (Jaiswal et al., 2022). However, our results failed to demonstrate such an association. This apparent paradox may reflect heterogeneity in patient populations, variation in baseline health status and comorbidity burden, differences in stroke subtypes, or suboptimal timing of vaccination relative to seasonal influenza activity.

Influenza vaccination can reduce the risk of vascular endothelial activation and thrombosis by inhibiting the inflammatory response, promoting the proliferation of smooth muscle cells and collagen in plaques, reducing lipid cores, and stabilizing atherosclerotic plaques (Bermúdez-Fajardo and Oviedo-Orta, 2011; Campbell and Rosenfeld, 2015). In addition, it can prevent infection severity and reduce sympathetic nerve excitation caused by the infection, which increases heart rate, blood pressure, and myocardial oxygen consumption. The above mechanisms can play a protective role in patients with CVD. Although some studies recommend administering high-dose influenza vaccine to the elderly and CVD patients, age-related immune alterations and disease-related immune dysfunction can weaken humoral responses, potentially reducing vaccine immunogenicity (Peikert et al., 2024; Van Ermen et al., 2013). High-dose influenza vaccines have demonstrated enhanced serologic responses in CVD patients compared with standard-dose formulations. However, our meta-analysis found no significant difference between high-dose and standard-dose influenza vaccination in preventing adverse cardiovascular outcomes among patients with established CVD or those at high cardiovascular risk. This finding may be explained by several factors. First, patients with CVD may have an impaired capacity to elicit robust humoral immune responses, and their elevated baseline risk for adverse events may attenuate the relationship between achieved antibody titers and clinical protection (Vardeny et al., 2009; Goudsmit et al., 2021). Second, the cardioprotective mechanisms of influenza vaccination may be mediated in part by pleiotropic anti-inflammatory effects that are independent of humoral immunity and thus not enhanced by higher antigen doses (Hjelholt et al., 2023). Although high-dose influenza vaccination did not demonstrate superior cardiovascular efficacy in our analysis, high-dose formulations did not increase complication rates compared to standard-dose vaccines. Therefore, these neutral findings should not alter current influenza vaccination guidelines that recommend high-dose formulations for high-risk populations, as such decision may still be justified based on enhanced immunogenicity and potential benefits for influenza-specific outcomes. Therefore, when formulating and promoting CVD prevention strategies, we recommend prioritizing public acceptance of the influenza vaccine and expanding influenza vaccination coverage rather than excessively focusing on dose optimization.

Our study has several limitations. First, the inherent constraints of the meta-analysis methodology that contributed to substantial heterogeneity across some outcomes are as follows: first, the core is the internal differences in study design. We combined RCTs with rigorous design and observational studies with low risk of bias that may have introduced residual confounding. Second, the included studies involved a diverse groups of populations across different countries and different periods, and their baseline cardiovascular risk and influenza epidemic strains during each study’s timeframe were different; in addition, the different research purposes of the different studies we included lead to the difference in main characteristics of the included population and their basic demographic information, and thus we cannot provide complete statistics, which contributed to the high heterogeneity. Third, the included observational studies were susceptible to residual confounding despite statistical adjustments. However, subgroup analyses demonstrated that the beneficial effects for CVM and MACE were more pronounced and statistically robust in RCTs than in observational studies, suggesting that these findings are less likely to be attributable to confounding. In addition, the shortest follow-up period in the existing studies was at least 1 year. However, for outcomes such as ACM and CVM, which require long-term follow-up, a 1-year follow-up period may introduce bias into our research results. In the future, more studies with longer follow-up periods and higher quality are needed to further refine and update our findings. We included studies reporting both ORs and HRs due to the specific characteristics of the available literature. Most studies focusing on our primary outcomes reported ORs, and the follow-up durations for these outcomes were relatively consistent. Only a subset of studies provided HRs. Combining different effect measures likely contributes to the substantial heterogeneity observed in our results. As more studies reporting HRs become available in the future, we should adopt a more rigorous approach to data collection when incorporating new literature to update our findings. Finally, Egger’s test we used has some limitations in the evaluation of publication bias. Although the results of funnel plot and Egger’s test suggest no obvious publication bias, the number of studies on some outcomes in this analysis is less than 10. The statistical power of Egger’s test to detect the asymmetry of the funnel plot is low, and there may be low potential publication bias. Moreover, the test results are sensitive to the heterogeneity of studies, while high heterogeneity is observed in some of our outcomes, which may affect the reliability of Egger’s test results (Egger et al., 1997; Sterne and Egger, 2001).

## 5 Conclusion

Our meta-analysis provides robust evidence supporting the cardiovascular benefits of influenza vaccination in patients with established CVD and in those at high cardiovascular risk. The high-dose formulations do not provide additional protection in reducing cardiovascular outcomes among patients with established CVD. These results underscore the importance of prioritizing vaccination uptake over dose selection in CVD prevention strategies.

## Data Availability

The original contributions presented in the study are included in the article/Supplementary Material; further inquiries can be directed to the corresponding authors.

## Supplementary material

SUPPLEMENTARY FIGURE S1 Quality assessment using the Cochrane risk-of-bias tool for randomized controlled trials (RCTs). **(A)** RCTs regarding the efficacy of influenza vaccination. **(B)** RCTs regarding the dosage administration of influenza vaccination.

SUPPLEMENTARY FIGURE S2 Forest plot of the primary outcomes in RCTs and cohort studies including **(A)** ACM and **(B)** MACE.

SUPPLEMENTARY FIGURE S3 Forest plot of the secondary outcomes in RCTs and cohort studies including **(A)** CVM, **(B)** ACH, and **(C)** HF.

SUPPLEMENTARY FIGURE S4 Forest plot of the secondary outcomes in RCTs and cohort studies including **(A)** MI and **(B)** stroke.

SUPPLEMENTARY FIGURE S5 Forest plot of the secondary outcomes in RCTs including **(A)** CVM, **(B)** ACH, **(C)** hospitalization for cardiorespiratory disease, **(D)** hospitalization for respiratory disease, **(E)** hospitalization for unstable angina, **(F)** MI, and **(G)** stroke.

SUPPLEMENTARY FIGURE S6 Funnel plots for publication bias for outcomes including **(A)** ACM, **(B)** ACH, **(C)** CVM, **(D)** HF, and **(E)** MACE.

SUPPLEMENTARY FIGURE S7 Sensitivity analysis using the leave-one-out method for primary outcomes including **(A)** ACM and **(B)** MACE.
