# Supplementary figures and images for: The benefits of influenza vaccination in patients with cardiovascular disease: a systematic review and meta-analysis
Source: Front Pharmacol. 2026 Jan 20;16:1701127. doi: 10.3389/fphar.2025.1701127 (PMC12865206; doi:10.3389/fphar.2025.1701127)

A

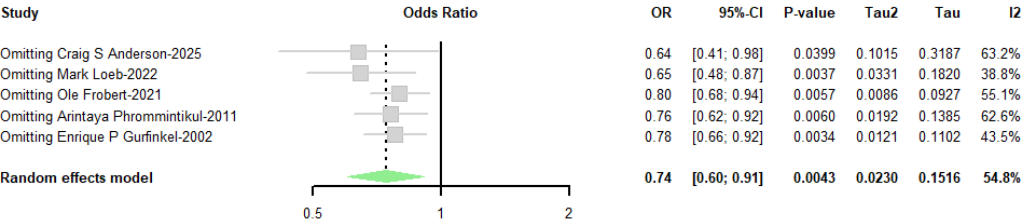

B

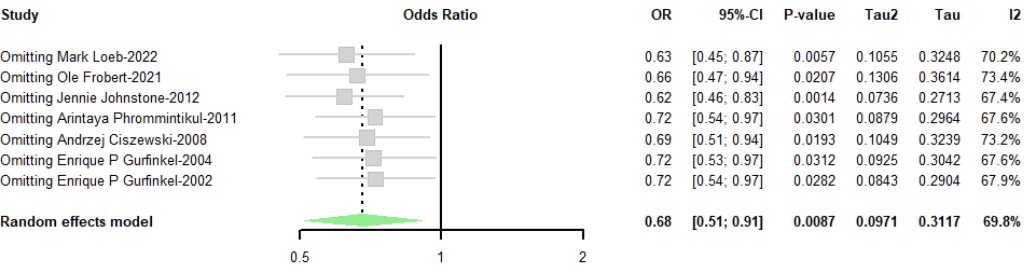

Supplement: Supplementary file 1 [file DataSheet7.PDF]

A

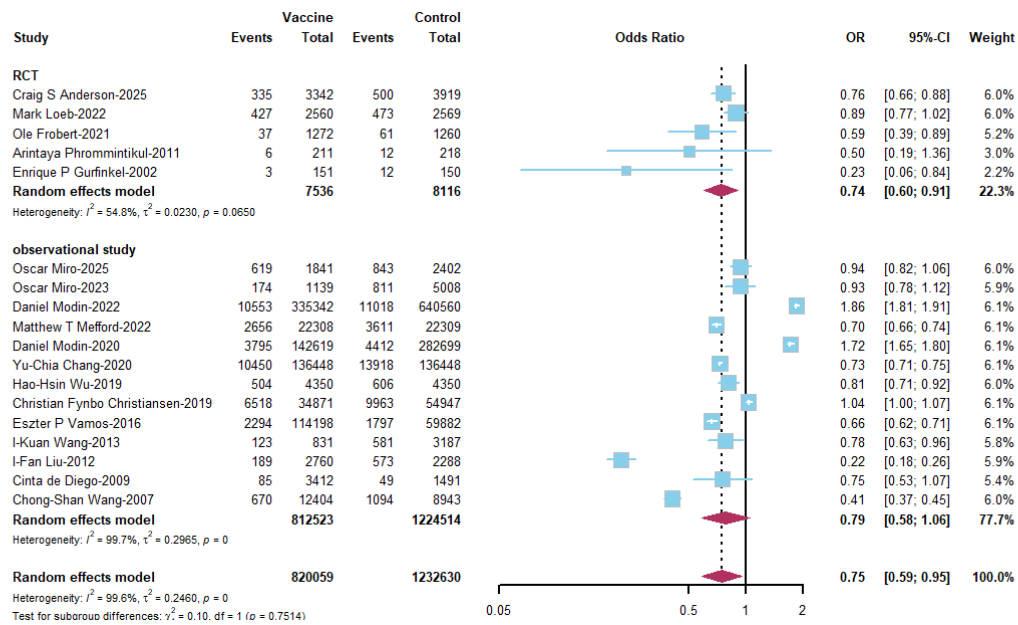

B

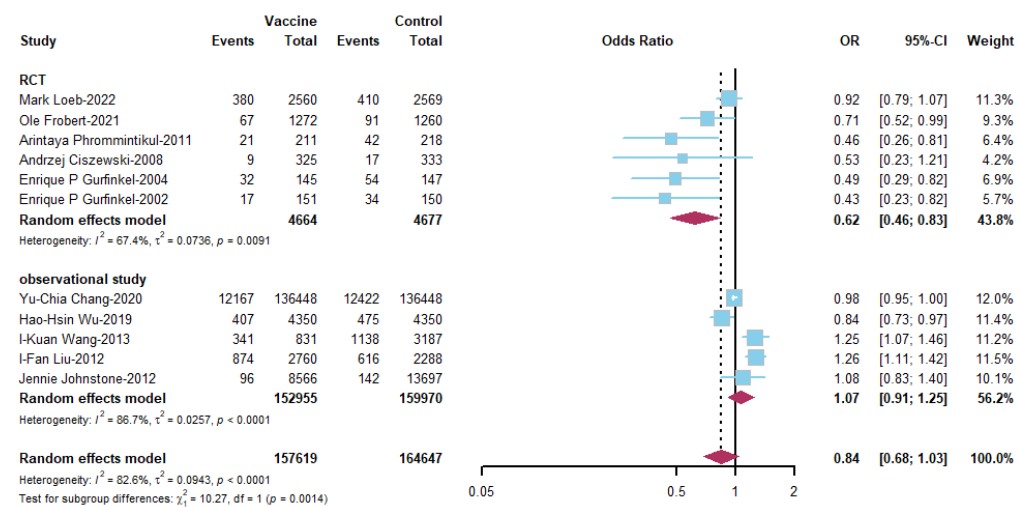

Supplement: Supplementary file 2 [file DataSheet2.PDF]

A

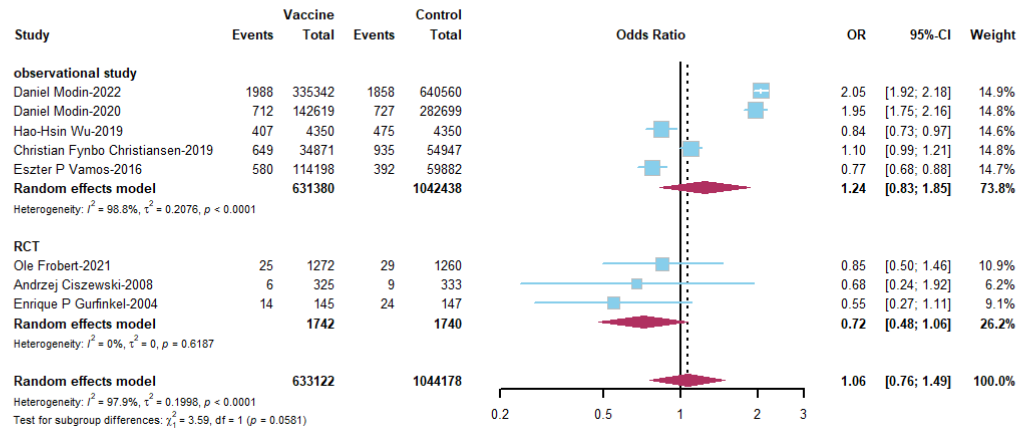

B

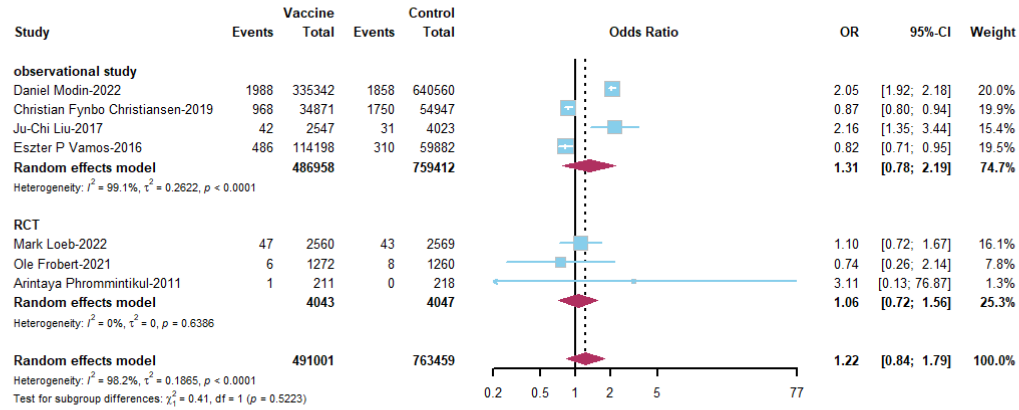

Supplement: Supplementary file 3 [file DataSheet4.PDF]

**A****Funnel Plot**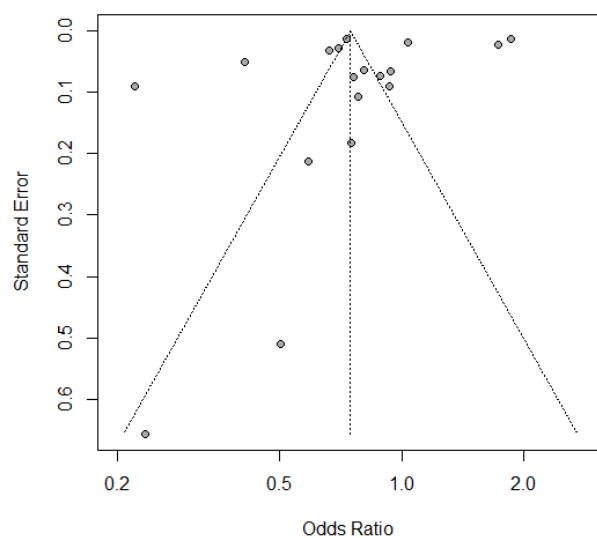**B****Funnel Plot**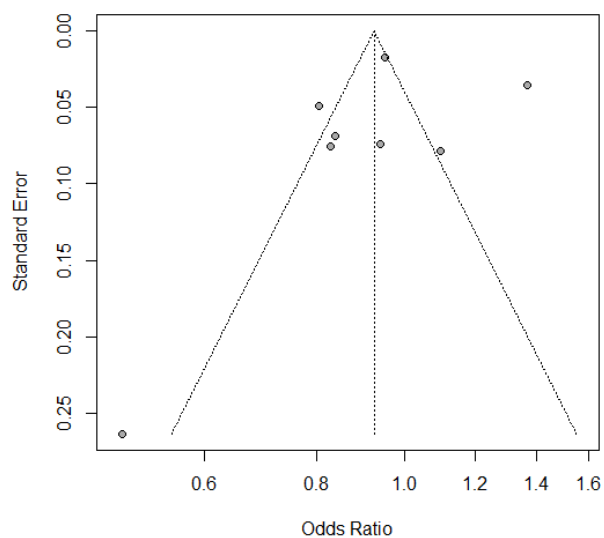**C****Funnel Plot**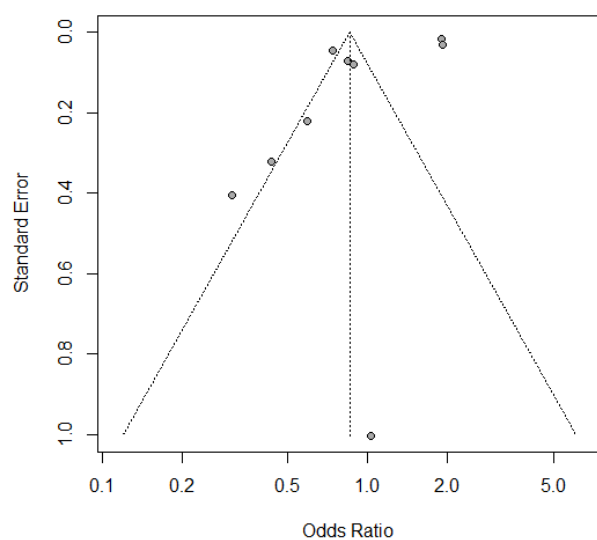**D****Funnel Plot**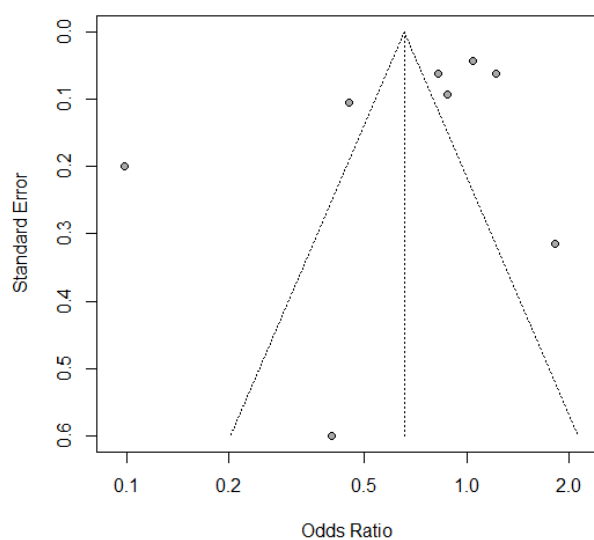**E****Funnel Plot**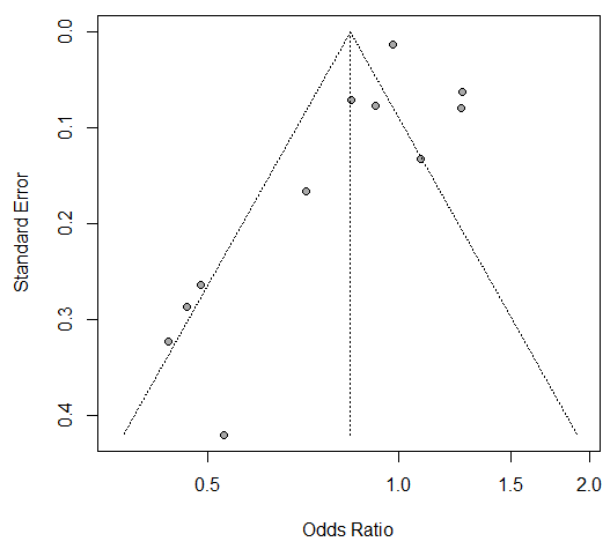

Supplement: Supplementary file 4 [file DataSheet6.PDF]

A

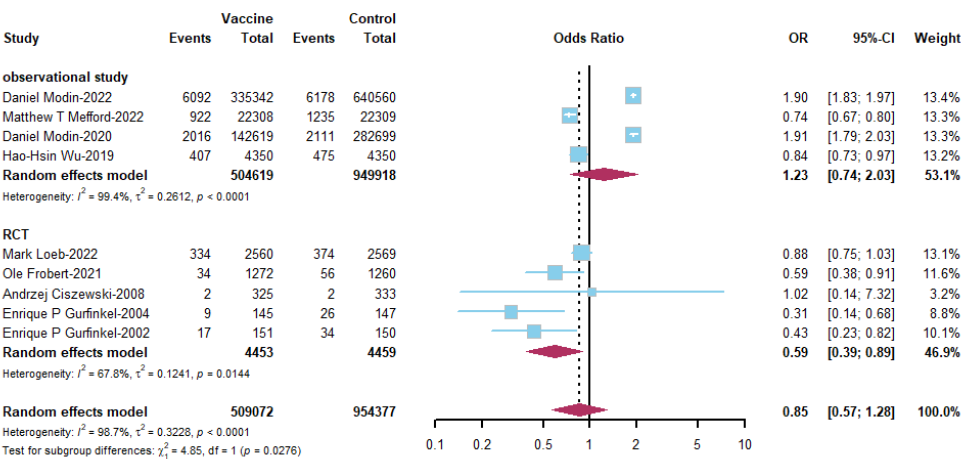

B

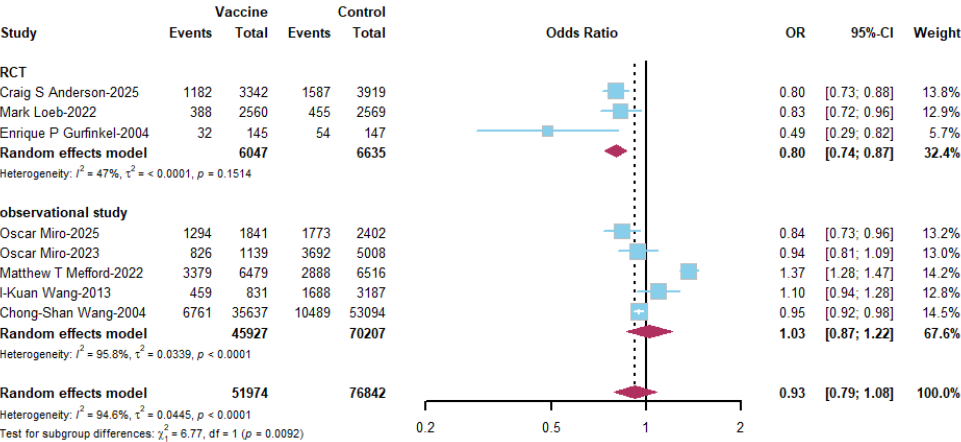

C

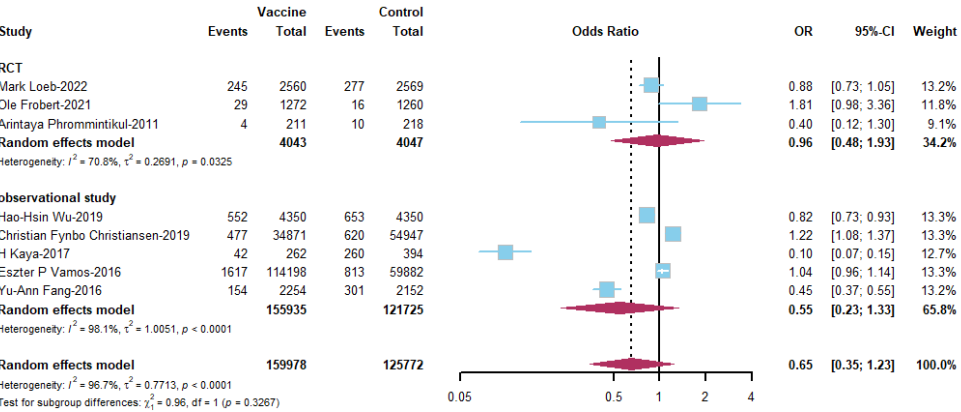

Supplement: Supplementary file 6 [file DataSheet3.PDF]

**A** Cochrane Risk of Bias Assessment

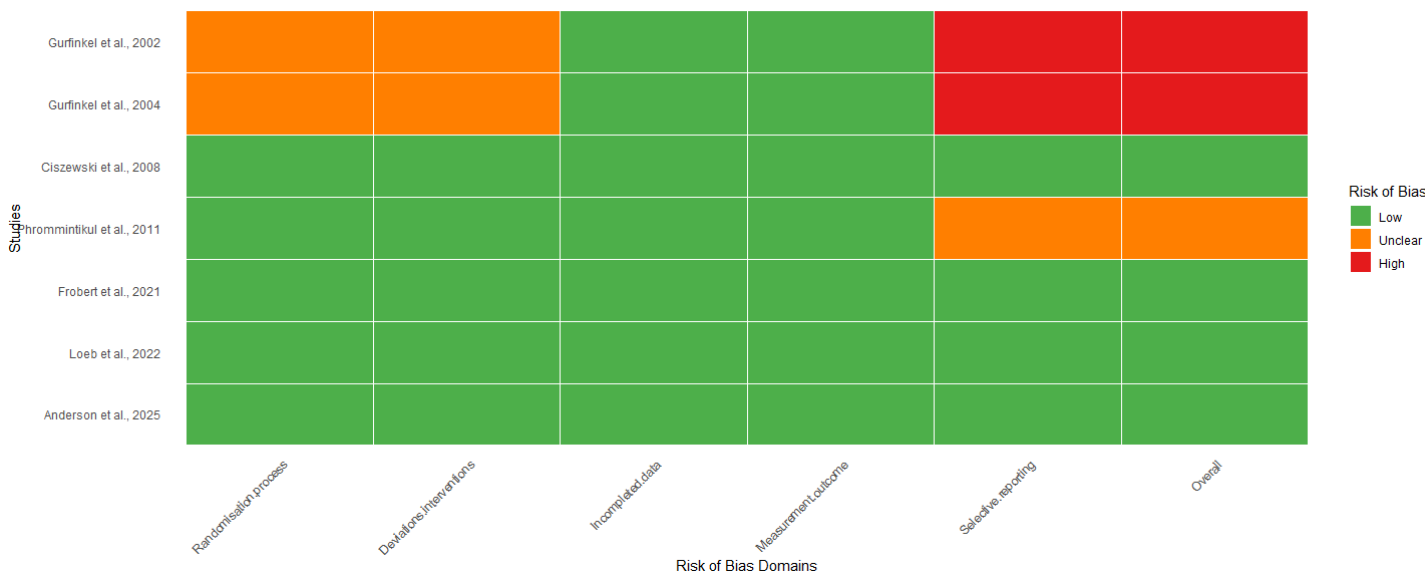

**B** Cochrane Risk of Bias Assessment

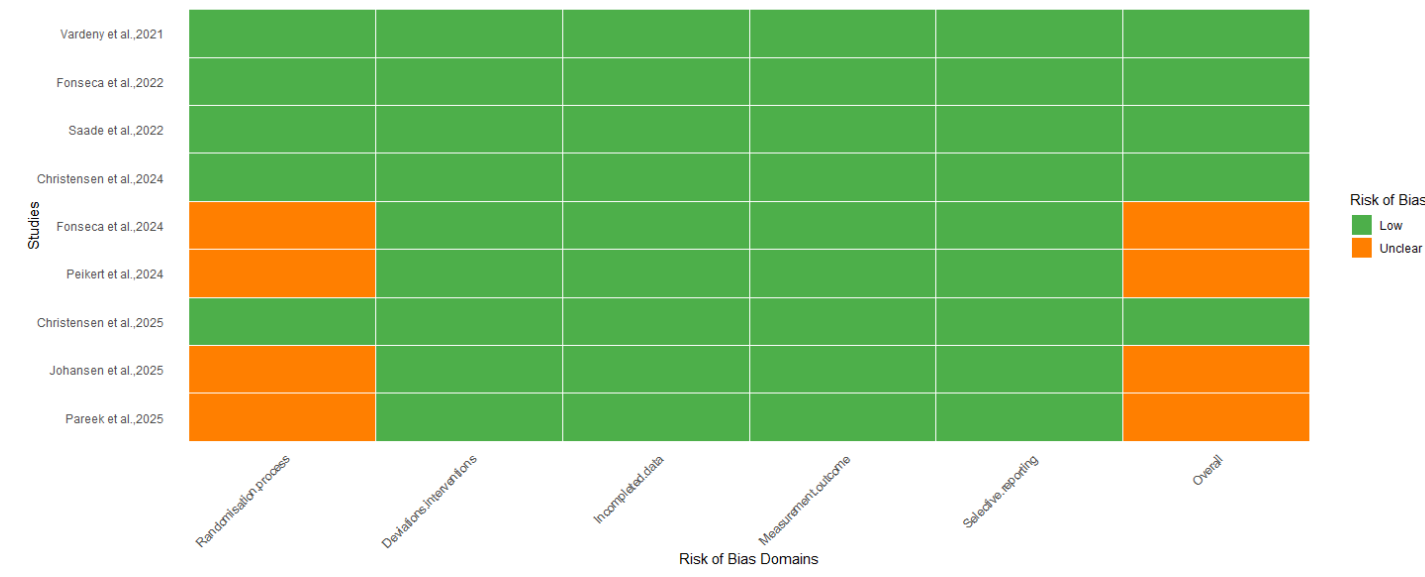

Supplement: Supplementary file 8 [file DataSheet1.PDF]

A

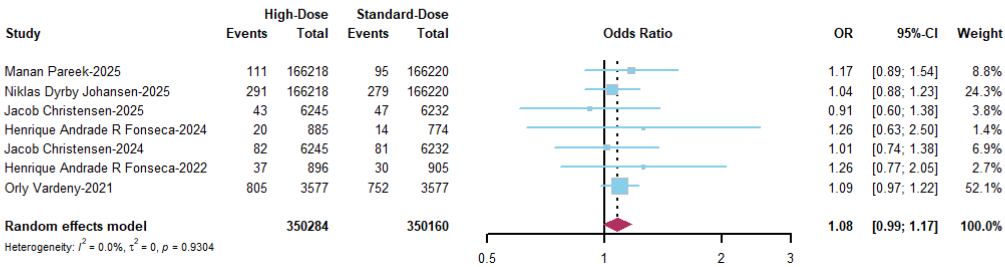

B

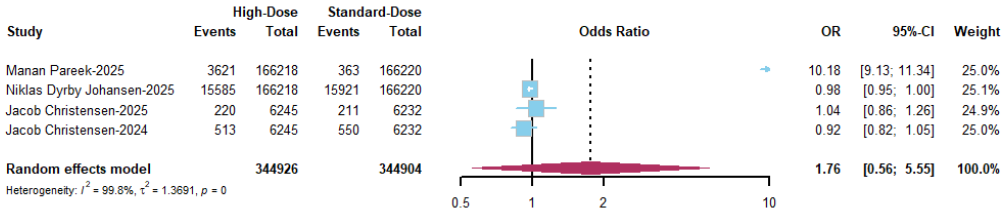

C

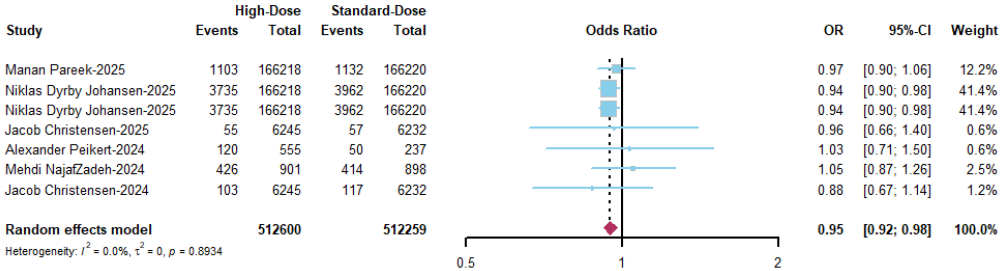

D

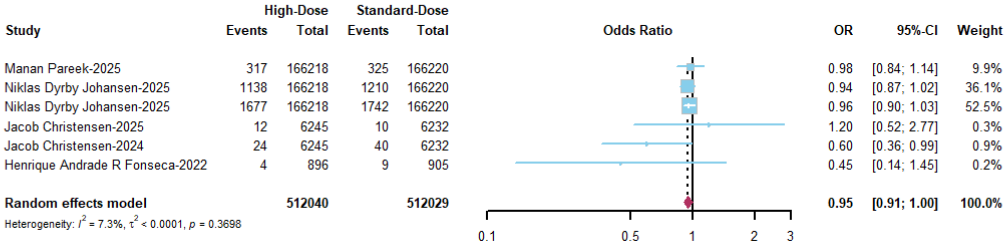

E

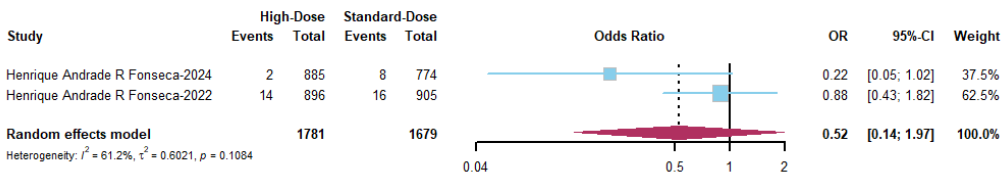

F

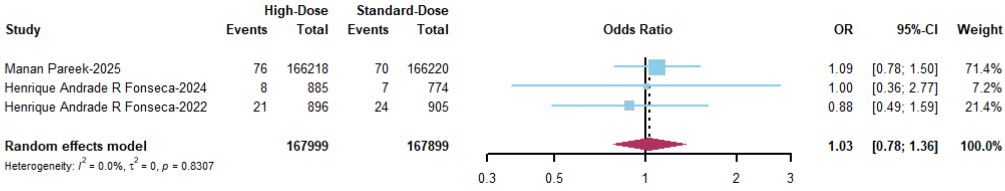

G

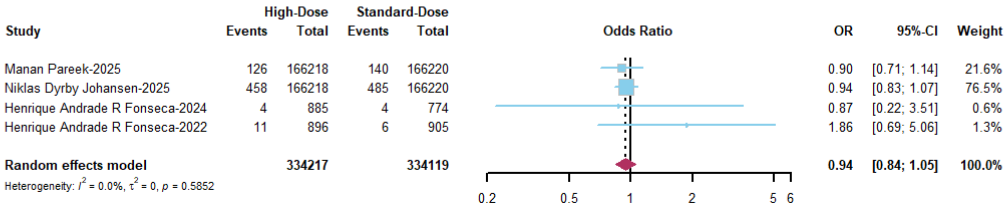

Supplement: Supplementary file 9 [file DataSheet5.PDF]
