# Supplementary material for: The benefits of influenza vaccination in patients with cardiovascular disease: a systematic review and meta-analysis
Source: Front Pharmacol. 2026 Jan 20;16:1701127. doi: 10.3389/fphar.2025.1701127 (PMC12865206; doi:10.3389/fphar.2025.1701127)
Supplement: Supplementary file 5 [file Table1.docx]

**Supplementary Table 1.** Search strategy

| **Electronic database** | **Search strategy** |
| --- | --- |
| **Pubmed** | (("influenza vaccines"[MeSH Terms] OR "influenza vaccine"[All Fields] OR "flu vaccines"[All Fields] OR "flu vaccine"[All Fields]) OR ("influenza"[MeSH Terms] OR "influenza"[All Fields] OR "flu"[All Fields] OR "influenza infection"[All Fields] OR "flu infection"[All Fields])) AND (("cardiovascular diseases"[MeSH Terms] OR "cardiovascular disease"[All Fields] OR "major adverse cardiovascular events"[All Fields] OR "MACE"[All Fields]) OR "mortality"[MeSH Terms] OR "mortality"[All Fields] OR "cardiovascular mortality"[All Fields] OR "heart failure"[MeSH Terms] OR "heart failure"[All Fields] OR "stroke"[MeSH Terms] OR "stroke"[All Fields] OR "myocardial infarction"[MeSH Terms] OR "myocardial infarction"[All Fields] OR "atrial fibrillation"[MeSH Terms] OR "atrial fibrillation"[All Fields] OR "acute coronary syndrome"[MeSH Terms] OR "acute coronary syndrome"[All Fields]) |
| **Embase** | (("influenza vaccines" OR "flu vaccines" OR "influenza vaccine" OR "flu vaccine" OR "influenza immunization" OR "flu immunization") OR ("influenza" OR "flu" OR "influenza infection" OR "flu infection" OR "human influenza")) AND (("cardiovascular diseases" OR "cardiovascular disease" OR "major adverse cardiovascular events" OR "MACE" OR "acute coronary syndrome" OR "ischaemic heart disease" OR "atrial fibrillation") OR ("mortality" OR "cardiovascular mortality" OR "sudden death") OR ("heart failure" OR "stroke" OR "myocardial infarction" OR "acute myocardial infarction")) |
| **Scopus** | TITLE-ABS-KEY(("influenza vaccines" OR "flu vaccines" OR "influenza vaccine" OR "flu vaccine" OR "influenza immunization" OR "flu immunization") OR ("influenza" OR "flu" OR "influenza infection" OR "flu infection")) AND TITLE-ABS-KEY(("cardiovascular diseases" OR "cardiovascular disease" OR "major adverse cardiovascular events" OR "MACE" OR "acute coronary syndrome" OR "atrial fibrillation") OR ("mortality" OR "cardiovascular mortality" OR "heart failure" OR "stroke" OR "myocardial infarction" OR "ischaemic heart disease")) |
