# Supplementary material for: The benefits of influenza vaccination in patients with cardiovascular disease: a systematic review and meta-analysis
Source: Front Pharmacol. 2026 Jan 20;16:1701127. doi: 10.3389/fphar.2025.1701127 (PMC12865206; doi:10.3389/fphar.2025.1701127)
Supplement: Supplementary file 7 [file Table2.docx]

**Supplementary Table 2.** Newcastle-Ottawa scale for quality assessment and bias assessment of observational studies.

| **Study** | **Selection** | | | | **Comparability** | **Outcome** | | | **Total*** |
| --- | --- | --- | --- | --- | --- | --- | --- | --- | --- |
|  | Representatives of the exposed group | Selection of non-exposed cohort | Ascertainment of exposure | Outcome of interest |  | Outcome assessment | Adequacy of follow-up duration | Adequacy of follow-up of cohort |  |
| Miró et al. 2025 | 1 | 1 | 1 | 1 | 2 | 1 | 1 | 1 | 9 |
| Miró et al. 2023 | 1 | 1 | 1 | 1 | 2 | 1 | 1 | 1 | 9 |
| Mefford et al. 2022 | 1 | 1 | 1 | 1 | 2 | 1 | 1 | 1 | 9 |
| Modin et al. 2022 | 1 | 1 | 1 | 1 | 2 | 1 | 1 | 1 | 9 |
| Modin et al. 2020 | 1 | 1 | 1 | 1 | 2 | 1 | 1 | 1 | 9 |
| Chang et al. 2020 | 0 | 1 | 1 | 1 | 2 | 1 | 1 | 0 | 7 |
| Wu et al. 2019 | 1 | 1 | 1 | 1 | 2 | 1 | 1 | 0 | 8 |
| Christiansen et al. 2019 | 0 | 1 | 1 | 1 | 2 | 1 | 1 | 1 | 8 |
| Liu et al. 2017 | 1 | 1 | 1 | 1 | 1 | 1 | 1 | 1 | 8 |
| Kaya et al. 2017 | 1 | 1 | 1 | 1 | 0 | 1 | 1 | 1 | 7 |
| Vamos et al. 2016 | 1 | 1 | 1 | 1 | 2 | 1 | 1 | 1 | 9 |
| Fang et al. 2016 | 1 | 1 | 1 | 1 | 2 | 1 | 1 | 1 | 9 |
| Wang et al. 2013 | 1 | 1 | 1 | 1 | 2 | 1 | 1 | 0 | 8 |
| Liu et al. 2012 | 1 | 1 | 1 | 1 | 0 | 1 | 1 | 1 | 7 |
| Johnstone et al.2012 | 1 | 1 | 1 | 1 | 2 | 1 | 1 | 0 | 8 |
| Diego et al. 2009 | 1 | 1 | 1 | 1 | 0 | 1 | 1 | 1 | 7 |
| Wang et al. 2007 | 1 | 1 | 1 | 1 | 2 | 1 | 1 | 0 | 8 |
| Wang et al. 2004 | 0 | 1 | 1 | 1 | 0 | 1 | 1 | 0 | 5 |
| NajafZadeh et al.2024 | 1 | 1 | 1 | 1 | 2 | 1 | 1 | 1 | 9 |

*Score >6 was considered as an adequate quality study.
